# Bacterial Community and Fermentation Quality of Ensiling Alfalfa With Commercial Lactic Acid Bacterial Additives

**DOI:** 10.3389/fmicb.2022.836899

**Published:** 2022-04-22

**Authors:** Na Na, Moge Qili, Nier Wu, Lin Sun, Haiwen Xu, Yi Zhao, Xiaobin Wei, Yanlin Xue, Ya Tao

**Affiliations:** ^1^Inner Mongolia Key Laboratory of Microbial Ecology of Silage, Inner Mongolia Engineering Research Center of Development and Utilization of Microbial Resources in Silage, Inner Mongolia Academy of Agriculture and Animal Husbandry Science, Hohhot, China; ^2^College of Foreign Languages, Inner Mongolia University of Finance and Economics, Hohhot, China; ^3^Inner Mongolia Youran Animal Husbandry Co., Ltd., Hohhot, China; ^4^Institute of Grassland Research, Chinese Academy of Agricultural Sciences, Hohhot, China

**Keywords:** alfalfa silage, bacterial community, fermentation quality, lactic acid bacterial additives, microbial counts, nutrition composition

## Abstract

The aim of this study was to determine the effects of six common commercial lactic acid bacteria (LAB) additives [A1, *Lactobacillus plantarum*, *L. buchneri*, and *Enterococcus faecalis*; A2, *L. plantarum* and *L. casei*; A3, *L. plantarum* and *L. buchneri*; A4, *L. plantarum*, *L. buchneri*, *L. casei*, and *Pediococcus acidilactici*; A5, *L. plantarum* (producing feruloyl esterase); and A6, *L. buchneri*, *P. acidilactici*, β-glucanase, and xylanase] on the bacterial community and fermentation quality of alfalfa silage. Alfalfa was harvested at the squaring stage, wilted in the field for 24 h, and ensiled without any additives (Control) or with A1, A2, A3, A4, A5, or A6. Microbial counts, bacterial community, fermentation parameters, and nutritional composition were determined after ensiling for 90 days. The total abundance of LAB genera on alfalfa pre-ensiling was 0.38% in bacterial community. The abundances of *Lactobacillus*, *Enterococcus*, and *Pediococcus* in the Control silage were 42.18, 40.18, and 8.09% of abundance, respectively. The abundances of *Lactobacillus* in A1-, A2-, A3-, A4-, and A5-treatments were 89.32, 92.93, 92.87, 81.12, and 80.44%, respectively. The abundances of *Pediococcus* and *Lactobacillus* in A6-treatment were 70.14 and 24.86%, respectively. Compared with Control silage, LAB-treated silage had lower pH and less ammonia nitrogen and water-soluble carbohydrates concentrations (*p* < 0.05). Further, the A5- and A6-treatments contained lower neutral detergent fiber, acid detergent fiber, and hemicellulose than other treatments (*p* < 0.05). Overall, LAB genera were presented as minor taxa in alfalfa pre-ensiling and as dominant taxa in alfalfa silage. Adding LAB additives improved the fermentation quality and altered the bacterial community of alfalfa silage. The main bacterial genera in Control silage were *Lactobacillus*, *Enterococcus*, and *Pediococcus*. *Lactobacillus* dominated the bacterial communities of A1-, A2-, A3-, A4-, and A5-treatments, while *Pediococcus* and *Lactobacillus* were dominant bacterial genera in A6-treatment. Inoculating A5 and A6 degraded the fiber in alfalfa silage. It is necessary to ensile alfalfa with LAB inoculants.

## Introduction

Ensiling has become a common and effective method for the long-term preservation of forage for livestock (Sun et al., 2021a). Silage enables anaerobic microbial fermentation to be dominated by lactic acid bacteria (LAB), which utilize water-soluble carbohydrates (WSC) to produce lactic acid (LA), reduce pH, and inhibit harmful microorganisms during ensilage process (Yang et al., 2020). Alfalfa (*Medicago sativa* L.) is a preferred perennial legume forage for livestock producers owing to its high nutritional value, especially its high crude protein (CP) concentration (Hartinger et al., 2019; Besharati et al., 2021; Netthisinghe et al., 2021). However, the second and third cuts of alfalfa in northern China are generally harvested during July and August, a period with an unreliable weather for alfalfa hay processing as it is in the rainy season. As a result, ensiling is the preferable method for conserving alfalfa during this period. Nevertheless, ensiling alfalfa with satisfactory fermentation quality is difficult because of the low dry matter (DM) and WSC concentrations and high buffering capacity (BC) (Sun et al., 2021b). Thus, wilting and applying additives to ensiled alfalfa are necessary to improve the fermentation quality and optimize microbial communities (Gao et al., 2021; Zhang et al., 2021).

Microbial composition, particularly LAB populations, plays a crucial role in the ensiling fermentation quality of silage (Bai et al., 2021). The development of next-generation sequencing technologies has helped to understand the differences in microbial communities and fermentation quality among silages (Wang C. et al., 2021). Previous studies revealed that inoculating LAB additives at ensiling alfalfa promotes bacterial community dynamics (especially *Lactobacillus* dynamics) during the fermentation process (Guo et al., 2018, 2020; Hu et al., 2020; Zhao S. et al., 2021). Other studies have also reported that *Lactobacillus* dominates the bacterial community in terminal alfalfa silage and in the mixing silage of alfalfa and whole-plant corn (Wang et al., 2020; Luo et al., 2021; Wang M. et al., 2021; Yang et al., 2021).

Inoculating LAB at ensiling optimizes the bacterial community and improves the fermentation quality of the terminal silage (Schmidt et al., 2009; Silva et al., 2016; Zhang et al., 2021; Zhao S. et al., 2021). Previous studies reported that the inoculation of ensiling alfalfa with self-screened LAB can promote the succession of *Lactobacillus* during the fermentation process and increase the abundance of *Lactobacillus* in terminal silage with good fermentation quality (Guo et al., 2018; Hu et al., 2020; Yang et al., 2021). Fermentation quality is improved in alfalfa silage treated with functional LAB screened to produce 3-phenyllactic acid (Wu et al., 2020), ferulic acid esterase (Su et al., 2019; Xie et al., 2021), and class IIa bacteriocin (Li et al., 2020). Sun et al. (2021b) revealed that alfalfa silage inoculated with LAB from ensiling material had greater fermentation quality than that inoculated with LAB from other forage sources. However, LAB screening has a low degree of commercialization, and the effect of common commercial LAB additives on fermentation quality and microbial communities of alfalfa silage has rarely been reported.

In the present study, six commercial LAB additives commonly used for ensiling alfalfa silage in northern China were collected. We hypothesized that the application of these additives at ensiling would improve the fermentation quality and optimize the bacterial community of alfalfa silage. The objective of this study was to determine the fermentation quality and bacterial community in alfalfa silage treated with commercial LAB additives.

## Materials and Methods

### Silage Preparation

Alfalfa was grown for 3 years on an experimental farm (40°46.265 N, 111°39.851E) at the Inner Mongolia Academy of Agricultural and Animal Husbandry Science, Hohhot, China, and harvested from four fields as replicates. The second-cut alfalfa was harvested at the squaring stage at 1 p.m. on June 1, 2019, and wilted in the fields for 24 h. The wilted forages from the four fields were separately chopped to 10–20 mm lengths using a chaffcutter (Hongguang Industry and Trade Co., Ltd., Zhejiang, China), thoroughly mixed, and then randomly divided into seven batches for seven treatments. After each additive (5 g) was mixed with distilled water (2,000 ml), the resulting mixture was allowed to rest for 2 h. The seven treatments were as follows: CK (control): 2 ml/kg fresh weight (FW) of distilled water; A1: 2 ml/kg FW of distilled water and 2 g/t FW (recommended amount, RA) of the first additive [*L. plantarum* LP28 (≥1.0 × 10^11^ CFU/g), *L. buchneri* LBC136 (≥1.0 × 10^9^ CFU/g), and *Enterococcus faecalis* EF08 (≥1.0 × 10^9^ CFU/g); Xinlaiwang I-HL for ensiling straw; Xinlaiwang Biotechnology Co., Ltd., Yangzhou, China]; A2, 2 ml/kg FW of distilled water and 2 g/t FW (RA) of the second additive [*L. plantarum* (≥6.0 × 10^10^ CFU/g) and *Lactobacillus casei* (≥4.0 × 10^10^ CFU/g); Xinlaiwang I for ensiling alfalfa. Xinlaiwang Biotechnology Co., Ltd., Yangzhou, China]; A3, 2 ml/kg FW of distilled water and 5 g/t FW (RA) of the third additive [*L. plantarum* 550 and 360 (≥1.3 × 10^10^ CFU/g) and *L. buchneri* 225 (≥7.0 × 10^9^ CFU/g); Zhuanglemei; Sichuan Gaofuji Biotechnology Co., Ltd., Chengdu, China]; A4, 2 ml/kg FW of distilled water and 1 g/t FW (RA) of the fourth additive [*L. plantarum*, *L. buchneri*, *L. casei*, and *Pediococcus acidilactici* (≥1.0 × 10^11^ CFU/g); BONSILAGE; Schaumann Agricultural Trading Co., Ltd., Shanghai, China]; A5, 2 ml/kg FW of distilled water and 1 g/t FW (RA) of the fifth additive [*L. plantarum* MF0932189 (producing feruloyl esterase, ≥ 1.0 × 10^11^ CFU/g); QXMG; Gansu Aopujintai Biological Engineering Co., Ltd., Lanzhou, China]; and A6, 2 ml/kg FW of distilled water and 1 g/t FW (RA) of the sixth additive [*L. buchneri* NCIMB 40788 (≥7.5 × 10^10^ CFU/g), *P. acidilactici* CNCM MA 18/5 M (≥5.0 × 10^10^ CFU/g), β-glucanase de *Aspergillus niger* MUCL 39199 (≥5,750 IU/g), and xylanase de *Trichoderma longibrachiatum* MUCL 39203 (≥30,000 IU/g), ≥ 1.25 × 10^11^ CFU/g; LaLSiL Dry; Lallemand Biotechnology Co., Ltd., Beijing, China]. After spraying distilled water with or without additives on chopped alfalfa and performing uniform mixing, approximately 500 g of forage was packed into a plastic bag (food grade, 300 mm × 400 mm; Qingye, Beijing, China) and sealed using a vacuum sealer (DZ-300; Qingye, Beijing, China). The bags were stored in a dark room for 90 days and then sampled for analysis. After sampling, the alfalfa pre-ensiling and silages were dried in a forced-air oven (BPG-9240A, Shanghai Yiheng Scientific Instrument Co., Ltd., Shanghai, China) at 65°C for 48 h, ground using a mill (FS-6D; Fichi Machinery Equipment Co., Ltd., Shandong, China) with a 1-mm screen, and dried in the same forced-air oven at 105°C until a constant mass was achieved. The dry matter (DM) content of the silages was corrected for the loss of volatiles during drying according to Weissbach and Strubelt (2008).

### Microbial Counts and Bacterial Community

The counts of LAB, coliforms, aerobic bacteria, and yeast were determined *via* culture on Man, Rogosa, Sharpe agar, violet red bile agar, nutrient agar, and potato dextrose agar, respectively, in an incubator (LRH-70, Shanghai Yiheng Science Instruments Co., Ltd., Shanghai, China) at 30°C for 72 h (Cai, 1999).

The bacterial communities of alfalfa pre-ensiling and silages were analyzed by Hangzhou Lianchuan Biotechnology Co., Ltd., Hangzhou, China, according to the method described by Sun et al. (2021a). The E.Z.N.A.^®^ Stool DNA Kit (D4015, Omega Inc., Norcross, GA, United States) was used to extract DNA from the bacteria according to the manufacturer’s instructions. Polymerase chain reaction (PCR) was carried out to amplify the V3–V4 region of the bacterial rRNA gene with primers 341F (5′-CCTACGGGNGGCWGCAG-3′) and 805R (5′-GACTACHVGGGTATCTAATCC-3′) (Logue et al., 2016), and the following cycling conditions: 98°C for 30 s, followed by 32 cycles of denaturation at 98°C for 10 s, annealing at 54°C for 30 s, and extension at 72°C for 45 s, and a final extension at 72°C for 10 min. The PCR products were purified using AMPure XT beads (Beckman Coulter Genomics, Danvers, MA, United States), quantified using Qubit (Invitrogen, Carlsbad, CA, United States), and then sequenced on an Illumina NovaSeq PE250 platform according to the manufacturer’s recommendations. High-quality clean tags were obtained from raw reads *via* quality filtering according to fqtrim (v0.94), and then filtered using Vsearch software (v2.3.4). Bacterial community diversity was calculated using QIIME2. Further, the sequence alignment of species annotation was performed using BLAST; the alignment databases were SILVA and NT-16S. Principal component analysis (PCA) of the bacterial community (at the genus level) of silages and bubble plot of the bacterial community (genus level) of silages were derived using R (version 3.2.1).^1^ Sequencing data were submitted to the NCBI Sequence Read Archive database (accession number: PRJNA744283).

### Fermentation Quality and Nutrition Composition

Fresh silage (25 g) was homogenized with sterile water (225 ml) using a flap-type sterile homogenizer (JX-05, Shanghai Jingxin Industrial Development Co., Ltd., Shanghai, China) for 100 s and filtered through four layers of cheesecloth to obtain the silage extract (Sun et al., 2021a). The pH of the silage extract was measured using a pH meter (PB-10; Sartorius, Gottingen, Germany). The organic acids [lactic acid (LA), acetic acid (AA), propionic acid, and butyric acid] were assessed using high-performance liquid chromatography (DAD, 210 nm, SPD-20A, Shimadzu Co., Ltd., Kyoto, Japan) and the following conditions: detector, SPD-20A diode array detector (210 nm); column, Shodex RS Pak KC-811 (50°C, Showa Denko K.K., Kawasaki, Japan); and mobile phase, 3 mM HClO_4_ (1.0 ml/min) (Bai et al., 2021). The concentrations of ammonia nitrogen (NH_3_-N) and total nitrogen (TN) were determined using the Kjeldahl method with a Kjeltec autoanalyzer (8400; Foss Co., Ltd., Hillerød, Denmark) (AOAC, 2005). Water-soluble carbohydrates (WSC) were assessed using anthrone-sulfuric acid colorimetry with a spectrophotometer (UV1102II, Shanghai Tianmei Scientific Instrument Co., Ltd., Shanghai, China), according to the method described by McDonald and Henderson (1964). The buffering capacity (BC) was assessed using acid-base titration, as described by Playne and McDonald (1966).

Crude protein (CP) concentration was calculated by multiplying the TN concentration by 6.25. Neutral detergent fiber (NDF) and acid detergent fiber (ADF) were assessed using an Ankom 2000 fiber analyzer (Ankom, Macedon, NY, United States) according to the method described by Van Soest et al. (1991). Hemicellulose concentration was calculated by the NDF concentration minus the ADF concentration. Crude ash was assessed using a muffle roaster (SX-4-10N, Shanghai Jingqi Instrument Co., Ltd., Shanghai, China) at 550°C for 5 h after carbonization.

### Statistical Analysis

The differences in microbial counts, sequencing data, alpha diversity, fermentation quality, and nutrition composition among treatments were analyzed with seven treatments and four repetitions using one-factor analysis of variance *via* the general linear model (GLM) procedure of SAS (version 9.1.3; SAS Institute Inc., Cary, NC, United States). The differences were compared using the least significant difference test, and significance was determined at *p* ≤ 0.05.

## Results

### Fermentation Quality and Nutrition Composition

The silage had lower pH and WSC concentration and higher BC content than fresh materials (*p* < 0.05; Table 1). The LAB-treatments had lower pH, NH_3_-N, and WSC than Control silage. Further, A2- and A6-treatments contained higher NH_3_-N than other LAB-treatments (*p* < 0.05). Compared with the Control silage and A1-treatment, the A4-, A5-, and A6-treatments contained lower AA (*p* < 0.05); A4- and A6-treatments had higher LA/AA (*p* < 0.05). The BC was the lowest in A4-treatment and the highest in A5-treatment among LAB-treatments (*p* < 0.05). No propionic and butyric acids were detected in alfalfa silages.

**TABLE 1:** Fermentation quality, water-soluble carbohydrates (WSC), and buffering capacity (BC) of alfalfa silages (*n* = 4).

Items	FM	CK	A1	A2	A3	A4	A5	A6	SEM	*p*-value
Ph	6.09a	4.70b	4.32c	4.36c	4.39c	4.36c	4.33c	4.38c	0.020	<0.001
LA (g/kg DM)	−	72.2	94.8	90.5	84.8	67.7	63.3	63.3	7.06	0.055
AA (g/kg DM)	−	51.1a	50.4a	40.0ab	30.6ab	20.2b	23.8b	19.8b	6.04	0.002
PA (g/kg DM)	−	ND	ND	ND	ND	ND	ND	ND	−	−
BA (g/kg DM)	−	ND	ND	ND	ND	ND	ND	ND	−	−
LA/AA	−	1.42c	2.07bc	2.52ab	2.78ab	3.38a	2.75ab	3.21a	0.222	<0.001
NH_3_-N (g/kg TN)	−	41.3a	23.2c	29.0b	19.2c	21.5c	23.5c	27.4b	1.1	<0.001
WSC (g/kg DM)	46.5a	15.1b	6.85c	6.54c	7.11c	5.79c	2.68c	4.50c	1.18	<0.001
BC (mEq/kg DM)	575d	858ab	842b	835b	838b	813c	867a	838b	5.87	<0.001

*Values with different letters indicate significant differences among fresh materials and silages. SEM, standard error of the mean; LA, lactic acid; AA, acetic acid; PA, propionic acid; BA, butyric acid; NH_3_-N, ammonia nitrogen; TN, total nitrogen; ND, not detected. FM, fresh materials; CK (Control), 2.00 ml/kg fresh weight (FW) of distilled water; A1, 2 g/t FW of the first additive and 2.00 ml/kg FW of distilled water; A2, 2 g/t FW of the second additive and 2.00 ml/kg FW of distilled water; A3, 5 g/t FW of the third additive and 2.00 ml/kg FW of distilled water; A4, 1 g/t FW of the fourth additive and 2.00 ml/kg FW of distilled water; A5, 1 g/t FW of the fifth additive and 2.00 ml/kg FW of distilled water; A6, 1 g/t FW of the sixth additive and 2.00 ml/kg FW of distilled water.*

The fresh material had a lower DM content than silages (*p* < 0.05; Table 2). The A4-treatment contained higher DM content than other treatments, with Control silage displaying a lower content than A3-treatment (*p* < 0.05). The NDF, ADF, and hemicellulose concentrations in A5- and A6-treatments were lower than those in other treatments and fresh materials (*p* < 0.05). The A3-tretment had higher NDF than Control silage and A1-treatment, with A1-treatment having a lower NDF than Control silage (*p* < 0.05). The A4-treatment contained the highest ADF, and A3-treatment had the greatest hemicellulose (*p* < 0.05). The crude ash concentration in A1-treatment was lower than that in A2- and A3-treatmetns (*p* < 0.05).

**TABLE 2:** Dry matter (DM, g/kg) and nutrition composition (g/kg DM) of alfalfa silages (*n* = 4).

Items	FM	CK	A1	A2	A3	A4	A5	A6	SEM	*p*-value
DM	485d	496c	503bc	506bc	512b	525a	507bc	504bc	2.69	<0.001
Crude protein	196	194	195	193	194	192	194	193	2.33	0.931
Neutral detergent fiber	375ab	359b	340c	373ab	384a	366ab	293d	294d	5.65	<0.001
Acid detergent fiber	230ab	236ab	220b	237ab	220b	245a	198c	201c	5.44	<0.001
Hemicellulose	145b	122c	119c	136bc	164a	121c	94.6d	93.3d	5.01	<0.001
Crude ash	98.2ab	97.0ab	94.5b	99.7a	99.5a	98.3ab	98.3ab	98.3ab	1.06	0.048

*Values with different letters indicate significant differences among fresh materials and silages. SEM, standard error of the mean. FM, fresh materials; CK (Control), 2.00 ml/kg fresh weight (FW) of distilled water; A1, 2 g/t FW of the first additive and 2.00 ml/kg FW of distilled water; A2, 2 g/t FW of the second additive and 2.00 ml/kg FW of distilled water; A3, 5 g/t FW of the third additive and 2.00 ml/kg FW of distilled water; A4, 1 g/t FW of the fourth additive and 2.00 ml/kg FW of distilled water; A5, 1 g/t FW of the fifth additive and 2.00 ml/kg FW of distilled water; A6, 1 g/t FW of the sixth additive and 2.00 ml/kg FW of distilled water.*

### Microbial Counts and Bacterial Community

The Control silage and A2-treatment had greater LAB count than other treatments and fresh materials (*p* < 0.05). Further, A1- and A3-treatments contained higher LAB count than A4-, A5-, and A6-treatments (*p* < 0.05), and the A4-treatment displayed the lowest LAB count (*p* < 0.05; Table 3). The aerobic bacterial count in fresh materials was higher than that in the silages (*p* < 0.05), and the aerobic bacterial count in Control silage was higher than that in LAB-treatments (*p* < 0.05). Moreover, the A4- and A6-treatments had lower yeast count than other treatments and fresh materials (*p* < 0.05). Coliforms were detected in fresh materials but not in silages.

**TABLE 3:** Microbial counts, sequencing data, and alpha diversity of bacteria in alfalfa silages (*n* = 4).

Items		FM	CK	A1	A2	A3	A4	A5	A6	SEM	*p*-value
Microbial counts (log CFU/g FW)	Lactic acid bacteria	5.59cd	7.29a	6.45b	7.09a	6.00bc	4.45f	5.36de	4.96e	0.157	<0.001
	Coliforms	5.31a	−	−	−	−	−	−	−	0.074	<0.001
	Aerobic bacteria	6.47a	5.55b	4.70c	4.55c	4.82c	4.24c	4.43c	4.77c	0.143	<0.001
	Yeasts	7.40a	7.25a	6.90a	7.49a	7.06a	4.91b	7.00a	5.19b	0.179	<0.001
Sequencing data	Raw reads	85,257	82,306	83,868	82,358	84,706	83,348	83,852	82,378	884	0.181
	Clean reads	71,561b	739,95ab	79,610a	766,55ab	78,702a	76,947ab	78,683a	77,120ab	1,384	0.007
Alpha diversity	Observed species	250ab	179ab	190ab	159ab	160ab	245ab	257a	147b	23.2	0.007
	Chao1	250ab	179ab	191ab	159ab	161ab	246ab	257a	147b	23.3	0.007
	Shannon	4.97a	3.34b	1.32ef	1.13f	1.86de	3.51b	2.56c	2.31cd	0.196	<0.001
	Simpson	0.904a	0.791a	0.291c	0.249c	0.534b	0.846a	0.559b	0.629b	0.035	<0.001
	Goods coverage	1.00	1.00	1.00	1.00	1.00	1.00	1.00	1.00	−	−

*Values with different letters indicate significant differences among fresh materials and silages. SEM, standard error of the mean. FM, fresh materials; CK (Control), 2.00 ml/kg fresh weight (FW) of distilled water; A1, 2 g/t FW of the first additive and 2.00 ml/kg FW of distilled water; A2, 2 g/t FW of the second additive and 2.00 ml/kg FW of distilled water; A3, 5 g/t FW of the third additive and 2.00 ml/kg FW of distilled water; A4, 1 g/t FW of the fourth additive and 2.00 ml/kg FW of distilled water; A5, 1 g/t FW of the fifth additive and 2.00 ml/kg FW of distilled water; A6, 1 g/t FW of the sixth additive and 2.00 ml/kg FW of distilled water.*

A total of 2,672,280 raw reads and 2,453,088 clean reads of the 16S rRNA gene were obtained from the 32 samples (Table 3). There were no differences in the number of raw reads among all silages and fresh materials (*p* > 0.05), and the fresh materials had lower clean reads than A1-, A3-, and A5-treatments (*p* < 0.05). More than 83,000 raw reads and 76,000 clean reads were derived for each sample.

The A5-treatment had higher observed species and Chao1 index than A6-treatment (*p* < 0.05; Table 3). Fresh materials had a higher Shannon index than silages (*p* < 0.05), and Shannon index in Control silage and A4-treatment were higher than those in the other treatments (*p* < 0.05). The Simpson index for the A3-, A5-, and A6-treatments was higher than that for the A1- and A2-treatments and lower than that for the fresh materials, Control silage, and A4-treatment (*p* < 0.05).

**FIGURE 1:**
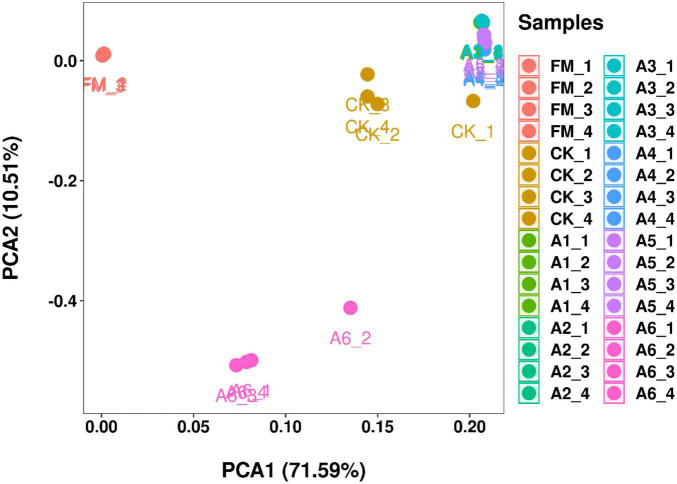
Principal component analysis (PCA) of the bacterial communities in silage and fresh materials (*n* = 4). FM, fresh materials; CK (Control), 2.00 ml/kg fresh weight (FW) of distilled water; A1, 2 g/t FW of the first additive and 2.00 ml/kg FW of distilled water; A2, 2 g/t FW of the second additive and 2.00 ml/kg FW of distilled water; A3, 5 g/t FW of the third additive and 2.00 ml/kg FW of distilled water; A4, 1 g/t FW of the fourth additive and 2.00 ml/kg FW of distilled water; A5, 1 g/t FW of the fifth additive and 2.00 ml/kg FW of distilled water; A6, 1 g/t FW of the sixth additive and 2.00 ml/kg FW of distilled water.

According to PCA, the bacterial communities of Control silage and A6-treatment were cleanly separated from each other and from other treatments (Figure 1). However, the A1-, A2-, A3-, A4-, and A5-treatments had aggregated bacterial community. In addition, the fresh materials contained a separate bacterial community from that of the silages.

**FIGURE 2:**
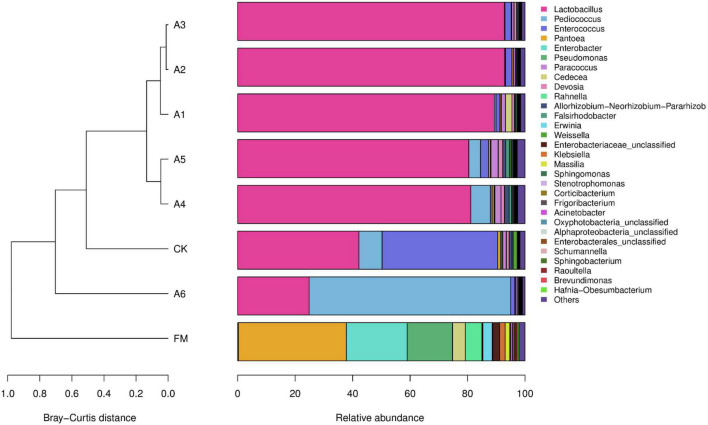
Relative abundance of the bacterial community (genus level) in silages and fresh materials (FM) (*n* = 4). FM, fresh materials; CK (Control), 2.00 ml/kg fresh weight (FW) of distilled water; A1, 2 g/t FW of the first additive and 2.00 ml/kg FW of distilled water; A2, 2 g/t FW of the second additive and 2.00 ml/kg FW of distilled water; A3, 5 g/t FW of the third additive and 2.00 ml/kg FW of distilled water; A4, 1 g/t FW of the fourth additive and 2.00 ml/kg FW of distilled water; A5, 1 g/t FW of the fifth additive and 2.00 ml/kg FW of distilled water; A6, 1 g/t FW of the sixth additive and 2.00 ml/kg FW of distilled water.

The most predominant bacterial genus in the A1-, A2-, A3-, A4-, and A5-treatments was *Lactobacillus*, with abundances of 89.32, 92.93, 92.87, 81.12, and 80.44%, respectively (Figure 2). The other main genera (>1%) were *Enterococcus*, *Cedecea*, and *Devosia* in A1-treatment; *Enterococcus* in A2- and A3-treatments; *Pediococcus*, *Paracoccus*, *Devosia*, and *Allorhizobium-Neorhizobium-Pararhizobium-Rhizobium* in A4-treatment; and *Pediococcus*, *Enterococcus*, *Paracoccus*, *Devosia*, *Allorhizobium-Neorhizobium-Pararhizobium-Rhizobium*, and *Falsirhodobacter* in A5-treatment. The main bacterial genera in Control silage and A6-treatment were *Lactobacillus* (42.18 and 24.86%, respectively), *Pediococcus* (8.09 and 70.14%, respectively), and *Enterococcus* (40.18 and 1.40%, respectively), followed by *Pantoea*, *Paracoccus*, and *Weissell*a in Control silage (>1%). Further, *Pantoea*, *Enterobacter*, and *Pseudomonas* were the dominant bacterial genera in fresh materials, with abundances of 37.59, 21.20, and 15.74%, respectively (Figure 2).

### Difference in Bacterial Community Among Silages and Fresh Materials

**FIGURE 3:**
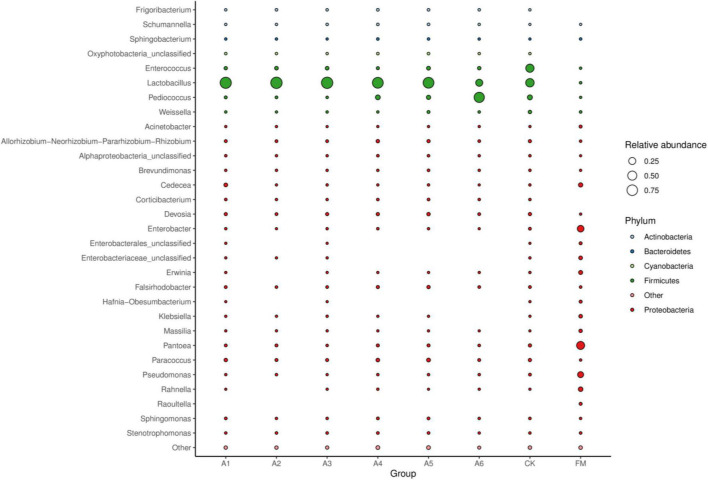
Bubble plot of the bacterial community (genus level) among silages and fresh material (*n* = 4, *p* < 0.05). FM, fresh materials; CK (Control), 2.00 ml/kg fresh weight (FW) of distilled water; A1, 2 g/t FW of the first additive and 2.00 ml/kg FW of distilled water; A2, 2 g/t FW of the second additive and 2.00 ml/kg FW of distilled water; A3, 5 g/t FW of the third additive and 2.00 ml/kg FW of distilled water; A4, 1 g/t FW of the fourth additive and 2.00 ml/kg FW of distilled water; A5, 1 g/t FW of the fifth additive and 2.00 ml/kg FW of distilled water; A6, 1 g/t FW of the sixth additive and 2.00 ml/kg FW of distilled water.

Compared with fresh materials, silages had higher *Lactobacillus*, *Pediococcus*, and *Enterococcus* (*p* < 0.05) and lower *Pantoea*, *Enterobacter*, *Pseudomonas*, *Cedecea*, and *Rahnella* (*p* < 0.05) (Figure 3). Control silage and A6-treatment contained less *Lactobacillus* than other treatments, with Control silage having higher than the A6-treatments (*p* < 0.05). The A6-treatment had higher *Pediococcus* than other treatments, with A1-, A2-, and A3-treatments displaying a lower than A4- and A5-treatments and Control silage (*p* < 0.05). Control silage had higher *Enterococcus* than other treatments (*p* < 0.05).

## Discussion

### Characteristics of Alfalfa Pre-ensiling

A previous study reported that ensiling alfalfa with satisfactory fermentation quality is difficult because of low LAB count, less WSC concentration, and higher BC in fresh forage (Sun et al., 2021b). In the present study, the epiphytic LAB count (5.59 log CFU/g FW, Table 3) in alfalfa pre-ensiling met the criteria (10^5^ log CFU/g FW) required for adequate fermentation (McDonald et al., 1991). However, the alfalfa pre-ensiling contained less WSC and moisture contents (46.5 g/kg DM and 515 g/kg) and higher BC (575 mEq/kg DM) (Tables 1, 2), resulting in higher pH (4.70), more NH_3_-N (41.3 g/kg), and lower LA/AA (1.42) in Control silage than those in LAB-treatments (Table 1). These suggest that it is necessary to ensile alfalfa with LAB additive for good fermentation quality. The LAB genera (*Weissella*, *Lactobacillus*, *Enterococcus*, and *Pediococcus*) were detected in alfalfa pre-ensiling with total abundance of 0.38% (Figure 2). Other studies found that the LAB genera have total abundance of less than 1.0% in alfalfa pre-ensiling (Hu et al., 2020; Zhao S. et al., 2021) and fresh whole-plant corn (Xu et al., 2019; Guan et al., 2020). The LAB genera were demonstrated to be generally presented as minor taxa in forage pre-ensiling. The main bacterial genera in alfalfa pre-ensiling were *Pantoea* (37.59%), *Enterobacter* (21.20%), and *Pseudomonas* (15.74%) (Figure 2). The findings agreed with those reported by Zhao S. et al. (2021) for high-moisture alfalfa [*Enterobacter* (33.93%), *Pseudomonas* (16.67%), and *Pantoea* (7.09%)]. However, other studies reported that the main bacterial genera (>10% of abundance) in alfalfa pre-ensiling were *Pseudomonas*, *Exiguobacterium*, and *Massilla* (Yang et al., 2020), Sphingobium (Hu et al., 2020), *Xanthomonas* and *Cyanobacteria* (Guo et al., 2020), and *Exiguobacterium* (Wang et al., 2019). The different bacterial communities in alfalfa pre-ensiling among those studies might be due to the differences in geographical locations (Wang C. et al., 2021).

### Fermentation Quality and Nutrition Composition of Silage

Ensiling alfalfa with LAB inoculants improves fermentation quality, as demonstrated by the increased LA content and decreased pH and NH_3_-N (Guo et al., 2020; Hu et al., 2020). In the present study, LAB inoculation at ensiling decreased pH and NH_3_-N in alfalfa silage. However, no difference in LA content was detected among all treatments, and the AA content in the Control silage and A1-treatment were higher than that in A4-, A5-, and A6-treatments. In addition, Control silage contained more WSC than LAB-treatments (15.1 vs. 2.86–7.11 g/kg DM); however, BC did not differ between Control silage and LAB-treatments (except A4) (Table 1). *Lactobacillus*, as the principal component of the additives used in the present study, was negatively correlated with WSC content in alfalfa silage (Supplementary Figure 1). The results suggest that other fermentation products (valeric acid, caproic acid, succinic acid, citric acid, ethanol, propanol, and 1,2-propandiol) might be generated during the ensiling process in LAB-treatments, and inoculating LAB at ensiling increased the utilization of WSC in silage during the fermentation process. This phenomenon was also observed in alfalfa silage (Xie et al., 2021), whole-plant corn silage (Jiang et al., 2020), and whole-plant sweet sorghum silage (Diepersloot et al., 2021). Inoculating heterofermentative LAB at ensiling reduces LA/AA in terminal silage by decreasing LA and increasing AA (Kung et al., 2018). The additives (except A5) used in the present study contained heterofermentative LAB (A1, *L. buchneri*; A2, *L. casei*; A3, *L. buchneri*; A4, *L. buchneri*, *L. casei*; A6, *L. buchneri*). However, A5-treatment had no difference in LA relative to other treatments; AA relative to A2-, A3-, A4-, and A6-treatments; and LA/AA relative to other LAB-treatments (Table 1). The finding might be due to homofermentative LAB dominating the fermentation process, as reflected by the total abundance of *L. plantarum*, *E. mundtii*, *E. faecium*, and *P. acidilactici* in Control silage and LAB-treatments (58.50, 85.50, 88.76, 73.21, 64.16, 74.42, and 77.28%, respectively) (Supplementary Table 1). Moreover, Guo et al. (2020) reported the undifferenced LA and AA concentrations between alfalfa silages treated with homo- and hetero-fermentative LAB.

During fermentation, proteolysis in silage is inevitable, owing to the presence of plant and microbial proteases (Thomas et al., 1980; Hassanat et al., 2007). The NH_3_-N, as part of the non-protein, shows the extent of silage preservation during the ensiling process, owing to its low utilization in the rumen (Xue et al., 2017; Yin et al., 2017). In the present study, NH_3_-N (41.3 g/kg TN) in Control silage was lower than the suggested concentrations in legume silage (< 120 g/kg TN) (Kung et al., 2018), indicating that the Control silage was well preserved. This finding might be because the higher DM content (480 g/kg) and the ideal anaerobic environment during the ensilage process cause a decrease in the activity of undesired microorganisms during ensiling. Propionic acid, butyric acid, coliforms, and *Clostridia* were not detected in any of the silages (Table 1 and Figure 2). Furthermore, compared with Control silage, the LAB-treatments displayed lower pH (4.70 vs. 4.32– to 4.39), NH_3_-N (41.3 vs. 19.2– to 29 g/kg TN), *Enterobacteriaceae* (2.43% vs. 0.1–% to 0.43%), and potentially pathogenic bacteria (6.50% vs. 1.05–% to 1.86%, expect A1) (Table 1 and Supplementary Figures 2, 3). This finding indicated that the fermentation products of the LAB additives used in the present study contributed more to the preservation of silage and the inhibition of undesired microorganisms during fermentation under low pH, low moisture, and ideal anaerobic conditions. Previous studies reported that LAB inoculation at ensiling decreased NH_3_-N content in alfalfa silage (Hu et al., 2020; Sun et al., 2021b).

In the present study, compared with Control silage, LAB-treatments had higher DM content, although the difference did not reach a significant level among Control silage and the A1-, A2-, A3-, and A6-treatments. Moreover, there were no differences in CP concentration among the silages (Table 2). Previous studies revealed that inoculating LAB at ensiling alfalfa increases the contents of DM and CP and improves the fermentation quality of terminal silage (Li et al., 2020; Wu et al., 2020). The results suggest that satisfactory fermentation quality contributes to increasing the DM content and preserving the CP of alfalfa silage. The concentrations of NDF, ADF, and hemicellulose in the A5- and A6-treatments were lower than those in the other treatments (Table 2), which might be related to the function of *L. plantarum* (producing feruloyl esterase) in A5 and the composition of A6 (β-glucanase and xylanase). Feruloyl esterase can promote cell wall degradation, especially in collaboration with cellulase and hemicellulose, by cleaving the ester or ether linkages between ferulic acid and sugars (Dilokpimol et al., 2016; Duan et al., 2021). Su et al. (2019) reported that inoculating feruloyl esterase-producing *Lactobacillus fermentum* at ensiling alfalfa decreased the NDF and ADF concentrations in terminal silage. β-glucanase, as one type of cellulase, can cleave glycosidic bonds in the amorphous regions of cellulose polymers (Takizawa et al., 2020). Moreover, xylanase contributes to the degradation of hemicellulose (Paës et al., 2012; Vucinic et al., 2021). Collectively, these findings indicate that A5 and A6, as additives, can degrade the cell wall during fermentation in alfalfa silage.

### Microbial Counts and Bacterial Community of Silage

Inoculating LAB at ensiling can increase LAB count in the terminal silage (Hu et al., 2020; Zhang et al., 2020). However, in the present study, the Control silage and A2-treatment displayed more LAB counts than the other treatments (Table 3). Moreover, the Control silage had the highest pH and aerobic bacterial count (Tables 1, 3). These results suggest that the LAB in the A2-treatment might have better resistance to less moisture and a low pH environment (494 g/kg and 4.26, respectively), and the microorganisms in the Control silage had greater activity under less moisture and weakly acidic conditions (504 g/kg and 4.70, respectively). The A4- and A6-treatments contained lower yeast counts than the other treatments (Table 3), which might be related to the presence of *P. acidilactici* in A4 and A6 as additives used in the present study. Previous studies reported that *P. acidilactici* inhibits effects on other microorganisms by producing antimicrobial bacteriocins (Kaya and Simsek, 2020; Surachat et al., 2021).

In the present study, most bacteria were detected in all samples the goods’ coverages reached approximately 1 (Table 3). The bacterial diversity of the silages was lower than that of the alfalfa pre-ensiling (Table 3). Furthermore, the material had a clearly separated bacterial community from the silages (Figure 1); similar results were reported by Zheng et al. (2017) and Zhao S. et al. (2021). This finding might be due to the large increasing abundance of LAB genera as the main bacterial taxa in silages (87.90%–95.30%) (Figures 2, 3). The Shannon and Simpson indexes for A4-treatment were higher than those for other LAB-treatments and did not differ from those of Control silage. Moreover, A1- and A2-treatments had lower Shannon and Simpson indexes than the other treatments (Table 3). These results suggest that the bacterial diversity was higher in the Control silage and A4-treatment, but lower in the A1- and A2-treatments. Interestingly, the same trend was detected in the number of main bacterial species, with > 10% abundance (Supplementary Figure 4). The bacterial communities in the Control silage and A6-treatment separated clearly from those of other treatments (Figure 1) due to the less abundance of *Lactobacillus* detected in the former (Figures 2, 3). Moreover, Control silage contained more *Lactobacillus* and *Enterococcus* and less *Pediococcus* than A6-treatment (Figures 2, 3), resulting in the separation of the bacterial communities between them (Figure 1).

Inoculation of LAB at ensiling optimizes the bacterial community and improves the fermentation quality of the terminal silage (Zhang et al., 2021; Zhao S. et al., 2021). In general, *Lactobacillus* dominates the bacterial community in well-preserved silage owing to its great capacity to produce acid and reduce pH during ensiling (Zi et al., 2021). In the present study, compared with Control silage, the LAB-treatments contained different bacterial communities (Figure 1) and had lower pH and NH_3_-N/TN (Table 1). Moreover, *Lactobacillus* dominated the bacterial communities in the A1-, A2-, A3-, A4-, and A5-treatments (89.32, 92.93, 92.87, 81.12, and 80.44%, respectively) (Figure 2). Nevertheless, the most dominant genus in A6-treatment was *Pediococcus* (70.14%) (Figure 2), which also caused lower pH, AA, and NH_3_-N/TN, and higher LA/AA than those for Control silage (Table 1). Such finding indicates that alfalfa silage is also well-preserved, with *Pediococcus* being the dominant genus. The difference in the most dominant bacterial genus among LAB-treatments might be related to the composition of the commercial additives used in the present study. *Lactobacillus plantarum* had the highest composition in A1, A2, A3, A4, and A5. Additionally, previous studies revealed that silage treated with *L. plantarum* had a greater abundance of *Lactobacillus* than Control silage (Zhang et al., 2020; Zhao C. et al., 2021). These results demonstrate that ensiling forage with *L. plantarum* increases the abundance of *Lactobacillus* in the terminal silage. Inoculating *P. acidilactici*, as one of the two components of A6 (*L. buchneri*, ≥ 7.5 × 10^10^ CFU/g; *P. acidilactici*, ≥ 5.0 × 10^10^ CFU/g), increased the abundance of *Pediococcus* in A6-treatment compared with other LAB-treatments (70.14% vs. 0.11–6.81%) (Figure 2). Moreover, the A4-treatment contained higher *Pediococcus* than A1-, A2-, A3-, and A5-treatments (6.81% vs. 0.11–4.07%), and *P. acidilactici* is one of the four components of A4. These results indicate that ensiling alfalfa with *P. acidilactici* increases the abundance of *Pediococcus* in the terminal silage. *Lactobacillus buchneri*, as one of the components of A1, A3, A4, and A6, was only detected in A6-treatment, with 0.17% abundance (Supplementary Table 1). Previous studies reported that *L. buchneri*, as an inoculant at ensiling, was detected as a minor taxon in alfalfa silage (Guo et al., 2020) and whole-plant corn silage (Xu et al., 2020; Netthisinghe et al., 2021). Moreover, *E. faecalis* (one of compositions of A1) was only detected in A1- and A2-treatments, with 0.025 and 0.001% abundances, respectively, and *L. casei* (one of compositions of A2 and A4) was only present in A6-treatment (0.011%) (Supplementary Table 1). These results indicate that *L. buchneri*, *L. casei*, and *E. faecalis* might have weaker competitiveness than *L. plantarum* and *P. acidilactici* in alfalfa silage with less moisture and low pH environments (475–497 g/kg and 4.33–4.39, respectively). The role of these LAB as the main components of LAB additives during the fermentation process in silage requires further study. The main bacterial genera in Control silage were *Lactobacillus* (42.18%), *Enterococcus* (40.18%), and *Pediococcus* (8.09%), indicating that the LAB genera dominated the bacterial community in Control silage (DM = 496 g/kg). Previous studies reported that the LAB population dominates the bacterial community in low-moisture alfalfa silage (DM > 400 g/kg) without any treatment (Guo et al., 2018, 2020) and presents as minor taxa in high-moisture alfalfa silage (DM < 270 g/kg) without any inoculants (Yang et al., 2020; Zhao S. et al., 2021). These results suggest that wilting alfalfa pre-ensiling may increase the total abundance of LAB genera in the bacterial community of alfalfa silage.

## Conclusion

The LAB genera are present as minor taxa in fresh alfalfa. Inoculating commercial LAB additives at ensiling alfalfa improved the fermentation quality, contributed to the preservation, and altered the bacterial community of the terminal silage. *Lactobacillus*, *Enterococcus*, and *Pediococcus* dominated the bacterial community in the Control silage. *Lactobacillus* was the most dominant bacterial genus in the A1-, A2-, A3-, A4-, and A5-treatments, and *Pediococcus* was the most dominant in A6-treatment. Further, addition of A5 and A6 decreased the concentrations of NDF, ADF, and hemicellulose in silage. Overall, the commercial lactic acid bacterial additives used in the present study can be employed to inoculate ensiling alfalfa in Northern China.

## Data Availability Statement

The datasets presented in this study can be found in online repositories. The names of the repository/repositories and accession number(s) can be found in the article/Supplementary Material.

## Author Contributions

NN, YX, and YT designed the study. NN wrote the manuscript. NN, MQ, NW, LS, YZ, and XW performed the experiments. HX, YX, and YT reviewed and edited the manuscript. NN, MQ, NW, HX, and YZ analyzed the data. YX and YT funded and supervised the experiments. All authors reviewed the manuscript.

## Conflict of Interest

XW and YT were employed by Inner Mongolia Youran Animal Husbandry Co., Ltd. The remaining authors declare that the research was conducted in the absence of any commercial or financial relationships that could be construed as a potential conflict of interest.

## Publisher’s Note

All claims expressed in this article are solely those of the authors and do not necessarily represent those of their affiliated organizations, or those of the publisher, the editors and the reviewers. Any product that may be evaluated in this article, or claim that may be made by its manufacturer, is not guaranteed or endorsed by the publisher.

## Footnotes

1
https://www.omicstudio.cn/tool

## Supplementary Material

Supplementary Figure 1 Redundancy analysis of the bacterial community (top 10 genera) and fermentation quality of alfalfa silage (*n* = 4).

Supplementary Figure 2 Relative abundance of the bacterial community (family level) in alfalfa silage (*n* = 4).

Supplementary Figure 3 Relative abundance of potentially pathogenic bacteria in alfalfa silage (*n* = 4).

Supplementary Figure 4 Relative abundance of the bacterial community (species level) in alfalfa silage (*n* = 4).

Supplementary Table 1 Relative abundance of lactic acid bacteria (species level) in alfalfa silage (*n* = 4).
