# Supplementary figures and images for: Bacterial Community and Fermentation Quality of Ensiling Alfalfa With Commercial Lactic Acid Bacterial Additives
Source: Front Microbiol. 2022 Apr 22;13:836899. doi: 10.3389/fmicb.2022.836899 (PMC9073077; doi:10.3389/fmicb.2022.836899)

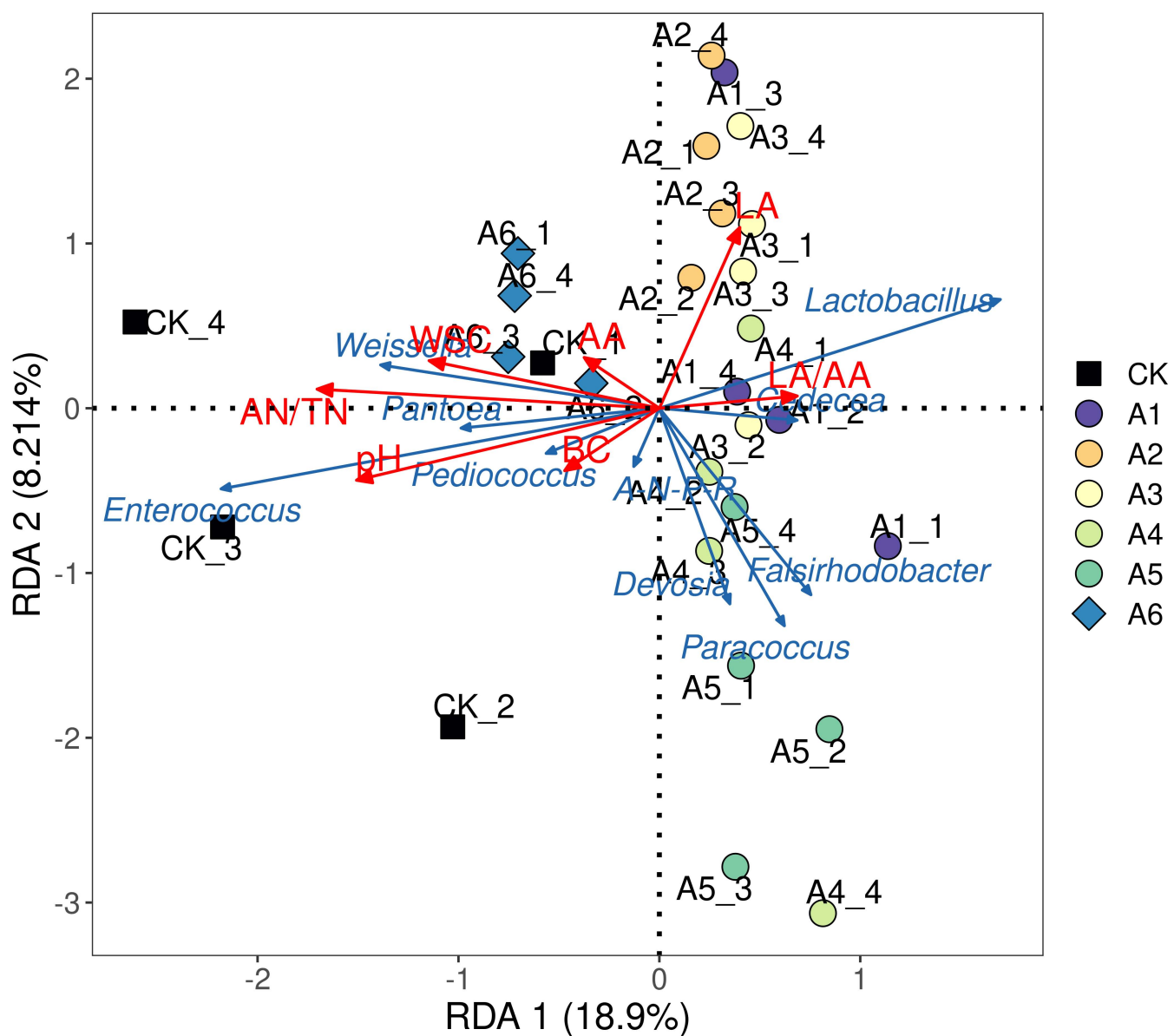

Supplement: Supplementary Figure 1 — Redundancy analysis of the bacterial community (top 10 genera) and fermentation quality of alfalfa silage (n = 4). [file Data_Sheet_1.PDF]

## Family

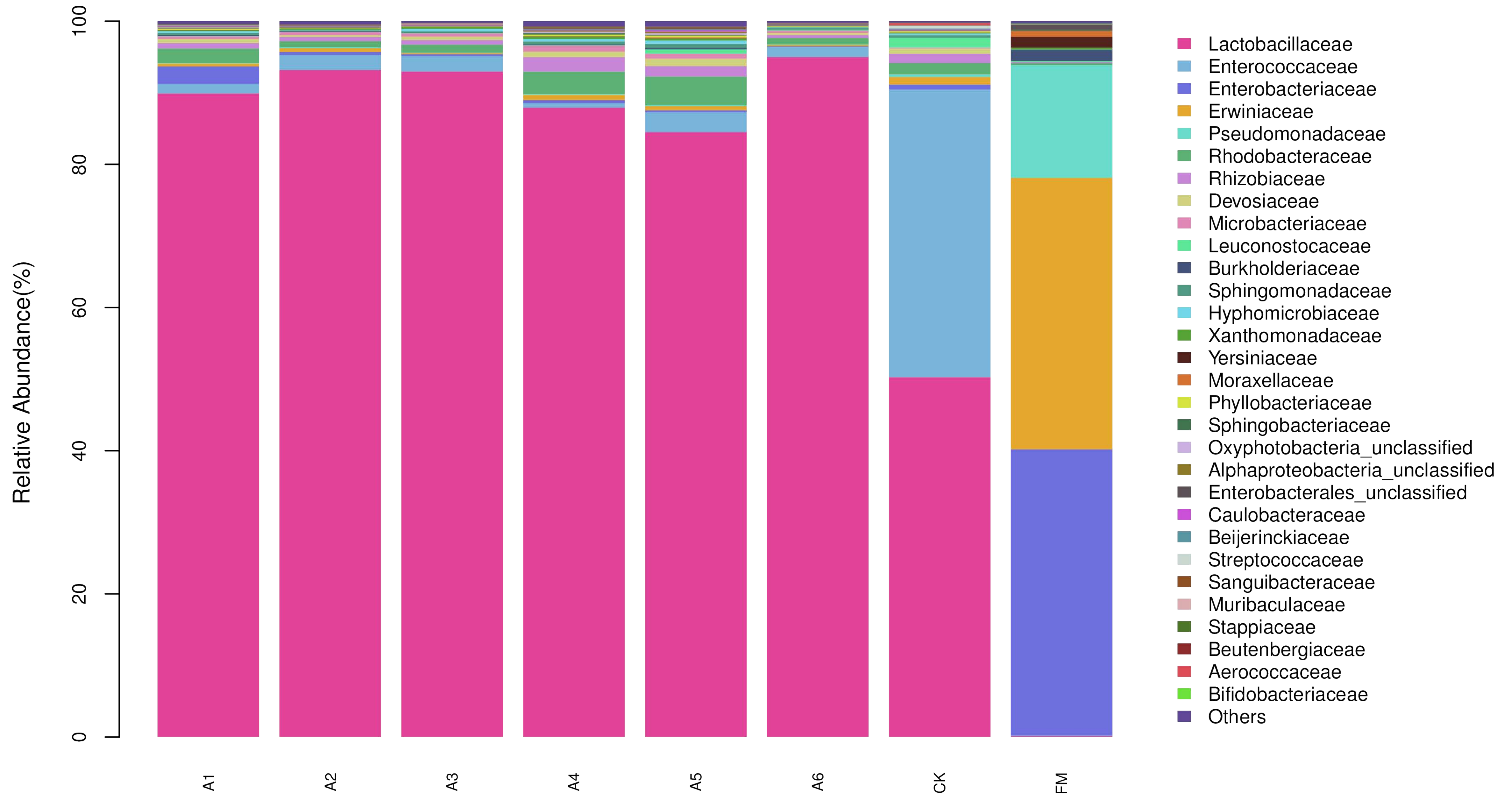

Supplement: Supplementary Figure 2 — Relative abundance of the bacterial community (family level) in alfalfa silage (n = 4). [file Data_Sheet_2.PDF]

Relative Abundance

1.0  
0.8  
0.6  
0.4  
0.2  
0.0

A1

A2

A3

A4

A5

A6

CK

FM

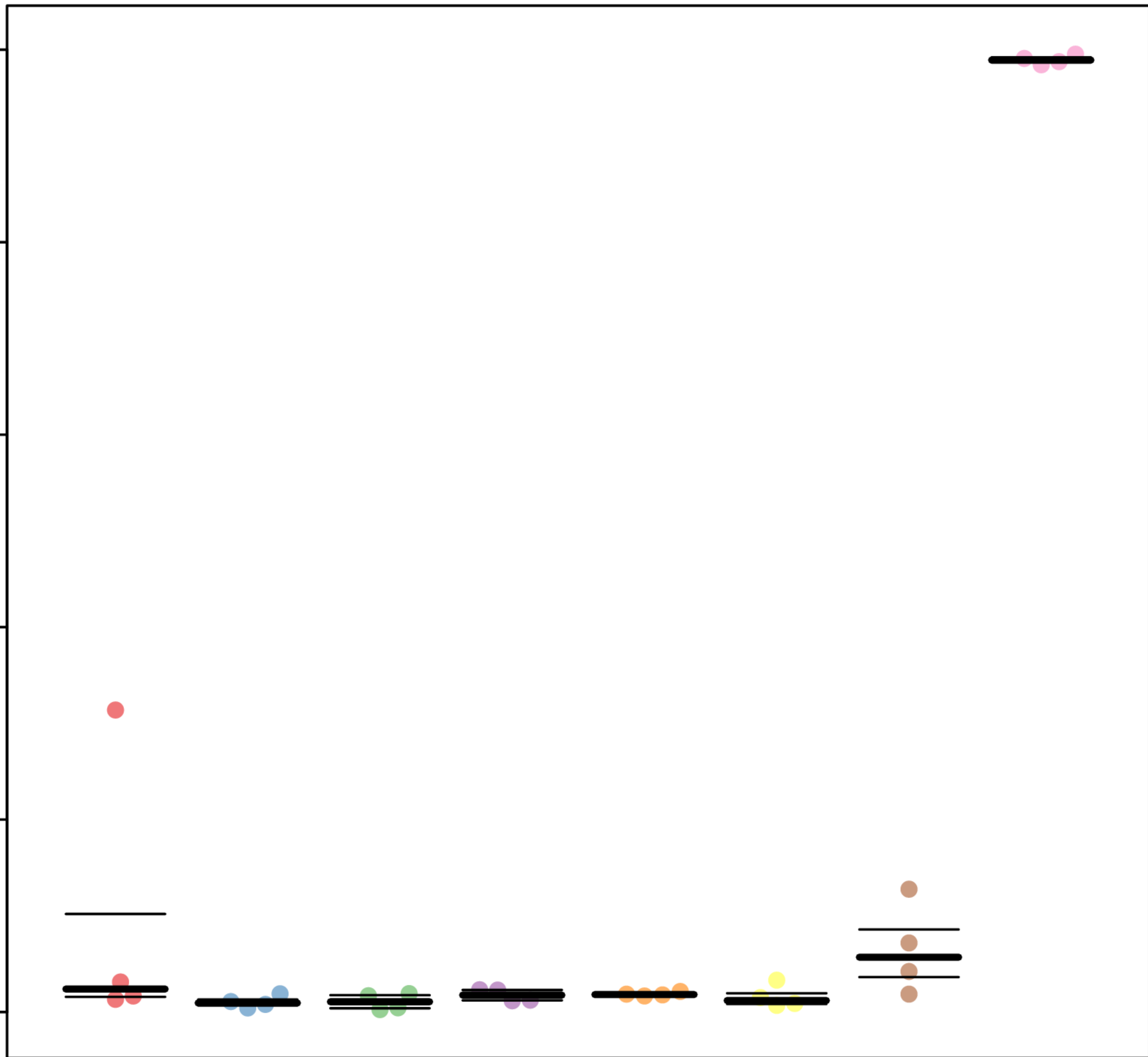

Supplement: Supplementary Figure 3 — Relative abundance of potentially pathogenic bacteria in alfalfa silage (n = 4). [file Data_Sheet_3.PDF]

## Species

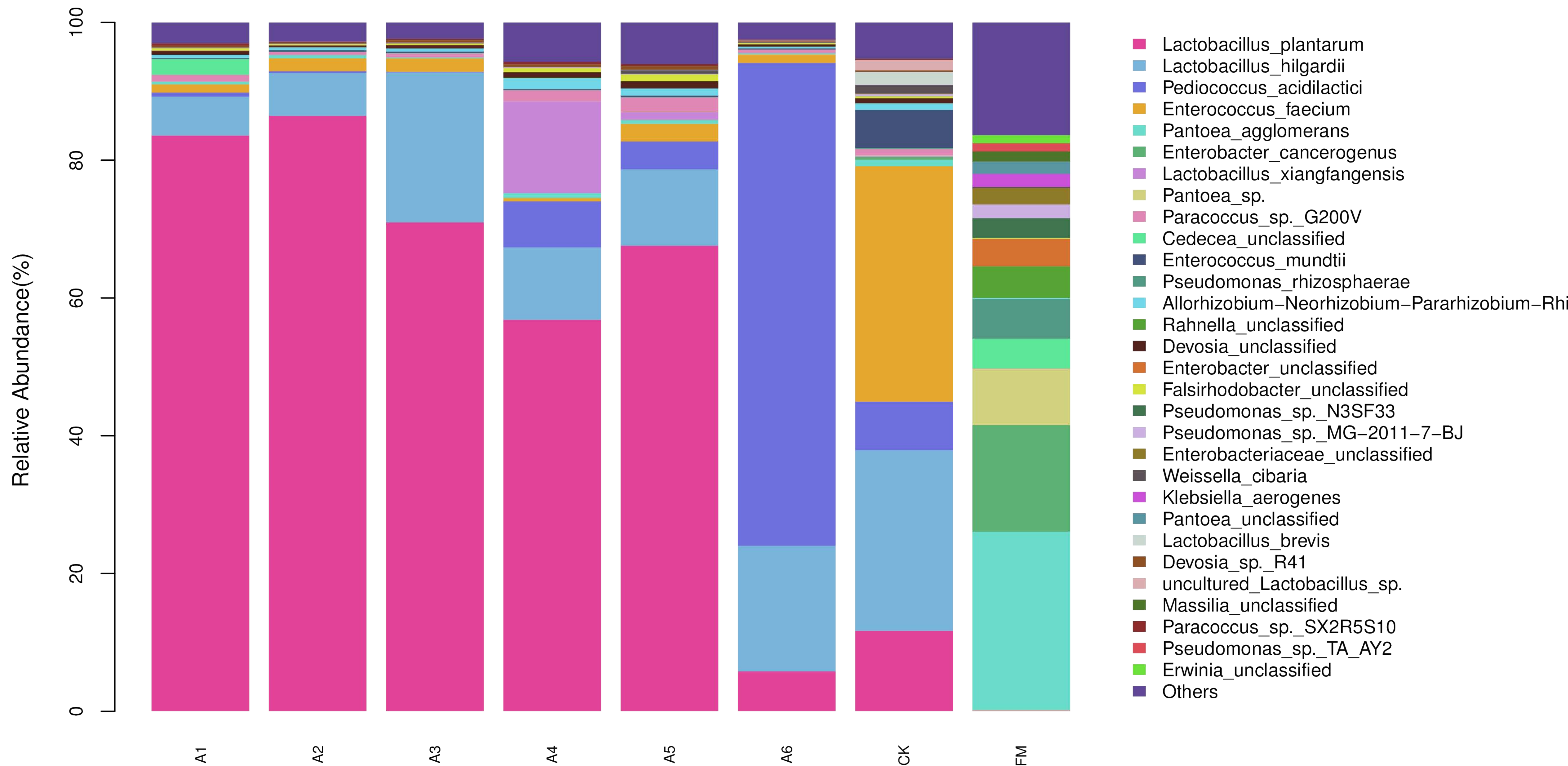

Supplement: Supplementary Figure 4 — Relative abundance of the bacterial community (species level) in alfalfa silage (n = 4). [file Data_Sheet_4.PDF]
